# Global Research Output and Theme Trends on Climate Change and Infectious Diseases: A Restrospective Bibliometric and Co-Word Biclustering Investigation of Papers Indexed in PubMed (1999–2018)

**DOI:** 10.3390/ijerph17145228

**Published:** 2020-07-20

**Authors:** Fan Li, Hao Zhou, De-Sheng Huang, Peng Guan

**Affiliations:** 1 School of Medical Informatics, China Medical University, Shenyang 110122, China; fanli@cmu.edu.cn; 2 Department of Epidemiology, School of Public Health, China Medical University, Shenyang 110122, China; zhouhao@cipuc.edu.cn (H.Z.); dshuang@cmu.edu.cn (D.-S.H.); 3 Department of Impression Evidence Examination Technology, Criminal Investigation Police University of China, Shenyang 110854, China; 4 Department of Mathematics, School of Fundamental Sciences, China Medical University, Shenyang 110122, China

**Keywords:** climate change, infectious diseases, bibliometric analysis, co-word analysis, biclustering, strategic diagram

## Abstract

Climate change is a challenge for the sustainable development of an international economy and society. The impact of climate change on infectious diseases has been regarded as one of the most urgent research topics. In this paper, an analysis of the bibliometrics, co-word biclustering, and strategic diagram was performed to evaluate global scientific production, hotspots, and developing trends regarding climate change and infectious diseases, based on the data of two decades (1999–2008 and 2009–2018) from PubMed. According to the search strategy and inclusion criteria, a total of 1443 publications were found on the topic of climate change and infectious diseases. There has been increasing research productivity in this field, which has been supported by a wide range of subject categories. The top highly-frequent major MeSH (medical subject headings)/subheading combination terms could be divided into four clusters for the first decade and five for the second decade using a biclustering analysis. At present, some significant public health challenges (global health, and travel and tropical climate, etc.) are at the center of the whole target research network. In the last ten years, “Statistical model”, “Diarrhea”, “Dengue”, “Ecosystem and biodiversity”, and “Zoonoses” have been considered as emerging hotspots, but they still need more attention for further development.

## 1. Introduction

It has been firmly established that the Earth is warming, which is shown by the increase in the average ocean temperature and air temperature, and in the melting of snow and ice. Global climate change is one of the most widely discussed topics, not only in the field of climate science or policy making, but also in a range of health researches [1,2]. It can affect human health via different pathways of complexity, directness, and scale [3,4,5]. A better understanding of the human health dimensions of climate change is necessary for protecting people from climate-sensitive hazards and the development of a sustainable coping strategy [6,7,8,9]. In particular, the direct and indirect impact of climate change on infectious diseases has been regarded as one of most urgent research topics [10,11,12]. It has been well accepted by the academic community that climate change could not only affect the pathogens’ ecology and the transmission dynamics of infectious diseases, but also the development of health promotion-related policy and the implementation process of the Sustainable Development Goals [13,14,15].

In the above-mentioned background, over the past several decades, there has been a large increase in scientific investigations about climate change and infectious diseases [14,15,16]. For example, the effect of global warming on vector-borne diseases, especially malaria, has been actively investigated [17,18,19,20,21,22]. The temperature can directly affect the biology of vectors and parasites, and increased precipitation may lead to an increase in the number and quality of breeding sites, and affect the availability of resting sites [23,24,25,26]. The temporal and spatial changes in climatic variables might affect the vectors, intermediate hosts, and, consequently, the risk of disease transmission [27,28]. Evidence has also indicated the impact of inter-annual and inter-decadal climate change on vector-borne diseases, which should be explored not only in a continental basis, but also in regional and local basis [29,30].

Bibliometrics is a kind of research method that analyzes bibliographic information using quantitative indicators, and has been widely employed for the statistical analysis of the bibliographic materials in a particular field [31,32]. In view of the impressive progress on climate change and infectious diseases, the quantitative and qualitative assessment of the scientific output will help to know the history, publication trends, research interest, and maturity degree of this field. Thus, the primary aim of the present work was to map the research output and theme trends in climate change and infectious diseases in the last 20 years (from 1999 to 2018), using the bibliometric indicators of production, word co-occurrence biclustering analysis, and strategic diagram. It is anticipated that this kind of reference can help the researchers in this field to prevent repeated work, avoid wasting resources, and know the research trends in the future. For the sake of shorthand, in the following section “this field” refers to “the field related to climate change and infectious diseases”.

## 2. Materials and Methods

### 2.1. Data Collection

The data from 1999 until 2018 were retrieved from PubMed of the National Library of Medicine on the web (http://www.ncbi.nlm.nih.gov/pubmed), with the medical subject headings (MeSH) terms “Climate Change”, “Climate”, “Meteorological Concepts”, “Weather”, and “Communicable Diseases”; the key words “Meteorological” and “Infectious diseases” in the title and abstract fields; and the Boolean combinations of these words as the retrieval strategy (for details of the retrieval strategy, see Table S1 in Supplementary Materials). The literature type was limited to journal articles. All publications were saved as two files, in the format of XML and MEDLINE, separately. Two independent researchers filtered the downloaded records manually according to the inclusion and exclusion criteria, after reviewing the titles and abstracts, and even, in some cases, the full text of the records. If they disagreed, the third person would judge whether a record was relevant. The included records were journal articles concerning both climate change and infectious diseases. The exclusion criteria were the following: (1) books, retracted publications, and bibliographies; (2) records of which the topic was related to political climate, social climate, economic climate, financial climate, organizational climate, etc.; and (3) repeated records.

Aiming to map the knowledge structure and theme trends of climate change and infectious diseases in the last 20 years, two periods of 10 years each were established, namely: 1 January 1999–31 December 2008, and 1 January 2009–31 December 2018. Furthermore, the comparative analysis for articles published in the two periods was conducted from the perspectives of bibliometric indicators and topics.

### 2.2. Data Analysis

Bibliographic Items Co-occurrence Matrix Builder (BICOMB), provided by Professor Cui from China Medical University [33], and Microsoft Excel were employed to determine the annual number of publications, most active journals, distribution of journals’ publication places, and the frequency of major MeSH/subheading combination terms. In the following section, “major MeSH/Subheading combination term” is referred to as “term” for short. The publication time of an article in this study was the final publication time, which meant that the information about the volume, pages, or serial article number had been released.

A research hotspot refers to a focus of research where researchers have conducted a lot of studies and published many related papers. By obtaining the frequencies and relationship of the words reflecting the content of the articles in a field, the hotspots of the field can usually be identified [34]. In this study, based on the principle for the g-index of the word frequency, a proper threshold (g) was set for the number of terms in order to generate a list of highly-frequent terms and a term-article matrix [35]. Egghe put forward a g-index used to reflect the contribution value of high-quality papers (i.e., highly cited papers) to a scientist. Similarly, a co-word analysis is used to select highly-frequent words to reflect the hotspots of a certain research field, so the g-index can also reflect the contribution value of highly-frequent words to all of the words in a given field [36]. Zhang et al. and Yang et al. have proved the simplicity and effectiveness of the g-index in selecting highly-frequent words in their empirical studies [35,37]. The method for the determination of the number, g, is as follows: firstly, all major MeSH/subheading combination terms were sorted in descending order of frequency; i was the sequence number of each term; when i was equal to g, the cumulative frequency of the first g terms was not less than g^2^, while that of the first (g + 1) terms was less than (g + 1)^2^. Then, the first g terms were considered as high-frequency terms [37]. If there were multiple terms with the frequency equal to that of the *g*th term, these terms were also identified as the highly-frequent terms. Next, a binary matrix with highly-frequent term-source article was created from BICOMB. By using the software “gCLUTO” (Graphical Clustering Toolkit, developed by Rasmussen et al. from University of Minnesota)) version 1.0 (University of Minnesota, Minneapolis, MN, USA), the matrix was imported for further biclustering [38]. The parameters in gCLUTO—repeated bisection for the clustering method, cosine for similarity function, and I^2^ for criterion function of clustering—were set based on those appropriate for the biclustering analysis of the literature. In order to gain the optimal number of clusters, the procedure for biclustering was repeated with different numbers of clusters. The biclustering result of the term-article matrix was presented through the visualization format of a mountain and matrix. With the aid of semantic relationships between the MeSH/subheading combination terms, and the content of the representative articles in each cluster, the basic framework of research hotspots on climate change-related infectious diseases was drawn and analyzed.

Moving forward, every hot research topic was put into the strategic diagram to show the relational patterns inside each cluster and among all of the clusters, so that the current status and evolutionary trends of this field could be revealed. In 1988, Law et al. proposed a strategic diagram to describe the internal linkages in the research domains and inter-domain interactions [39]. The strategic diagram is manifested as a two-dimensional chart, with the horizontal axis representing the centrality (the average value of external links, and external links refer to the sum of times that every term in a given cluster and every term in other clusters co-occur in the same article) and the vertical axis standing for the density (the average value of internal links, and internal links are the sum of times that every pair of terms in a given cluster co-occur in the same article) [40]. The centrality is used to judge the degree to which each term is connected with the terms in the other clusters, which can indicate the degree that one theme affects the others. The greater the number and intensity of links between a subject domain and other subject domains, the more central the subject domain becomes in the whole research work. The density is used to measure the closeness degree of the internal terms inside the same cluster, indicating the strength of relations that make terms into a cluster, i.e., the ability of one theme to maintain and develop itself [41]. Based on the results of the co-word clustering analysis and co-occurrence matrix of the highly-frequent terms, the density and the centrality were calculated for each cluster. The origin of coordinate is the average value of all centralities and that of all densities. With the help of the content, as well as the centrality and density of each cluster, the development status of the hot research topics in the two decades was presented by strategic diagrams, from which the evolutionary trend of the global research on climate change and infectious diseases was analyzed and discussed.

## 3. Results

### 3.1. Growth and Journals of the Relevant Publications

**Figure 1:**
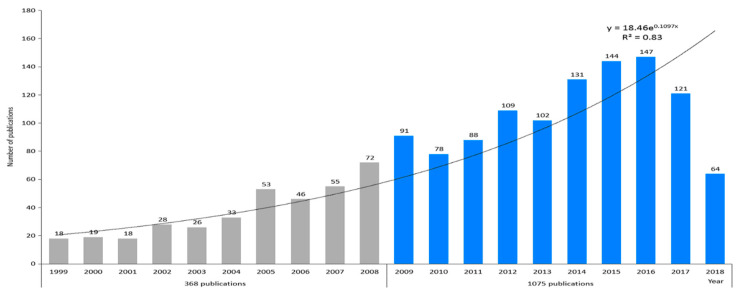
Temporal distribution of research output about climate change and infectious diseases (PubMed sourced).

Based on our search strategy and on the inclusion and exclusion criteria, 1443 journal articles were retrieved in PubMed on the topic of climate change and infectious diseases from 1999 to 2018. The annual number of related articles grew exponentially from only 18 in 1999, to the maximum, 147 in 2017, as shown in Figure 1, where an exponential trend line could be added (the degree of fitting, R^2^, is 0.83). For the two periods, 1999–2008 and 2009–2018, there were 368 and 1075 journal articles involved, respectively, which were then subjected to a comparative analysis.

**Figure 2:**
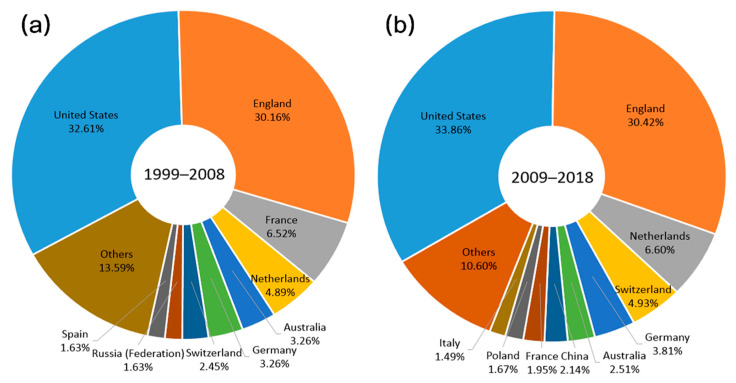
Publication places of journals publishing articles on climate change and infectious diseases. (**a**) Publication places of journals publishing relevant articles from 1999 to 2008; (**b**) publication places of journals publishing relevant articles from 2009 to 2018.

Altogether, 521 journals were involved in the field (1999–2008: 226 journals; 2009–2018: 407 journals). The United States and England were always two major publication places of journals publishing relevant articles in the two decades, as illustrated in Figure 2. The third publication places were France in the first decade and the Netherlands in the second decade. Table 1 displays the top ten productive journals for each period, as well as their languages, publication places, and number of publications. In 1999–2008, the top three most active journals were *Emerging Infectious Diseases*, *Journal of Travel Medicine*, and *Annals of the New York Academy of Science*, whereas in the latter ten years, *PLoS One*, *International Journal of Environmental Research and Public Health*, and *Epidemiology and Infection* were the most popular.

### 3.2. Highly-Frequent MeSH/Subheading Combination Terms and Their Cluster Pattern

From the articles included, 26 and 39 high-frequency major MeSH/subheadings combination terms were extracted for the first and second decade, respectively, based on the method for the g-index of the word frequency mentioned above, with a cumulative frequency percentage of 32.95% and 37.39%, respectively (Tables S2 and S3 in Supplementary Materials). Furthermore, these terms were subject to a co-word biclustering analysis to reveal the research hotspots for climate change and infectious diseases in the past two decades.

**Figure 3:**
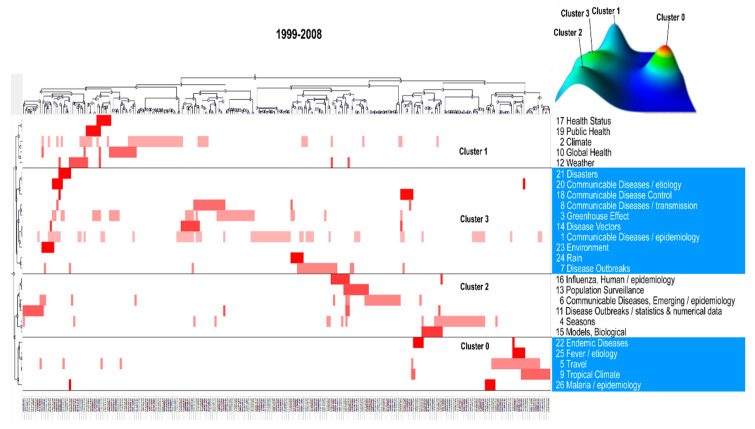
Mountain and matrix visualization of biclustering of highly-frequent major medical subject headings (MeSH)/subheading combination terms and articles on climate change and infectious diseases from 1999 to 2008.

**Figure 4:**
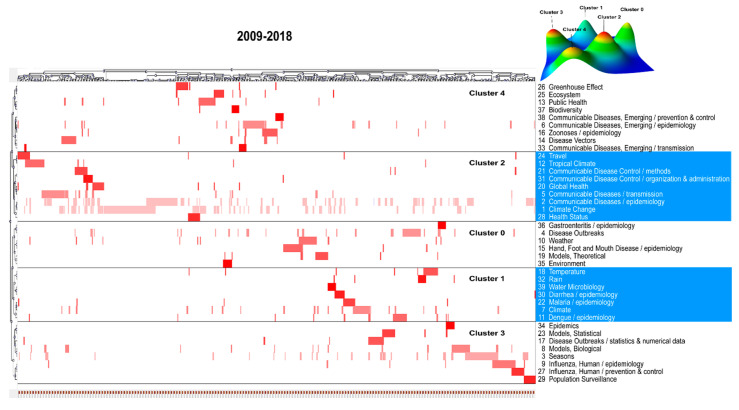
Mountain and matrix visualization of biclustering of highly-frequent major MeSH/subheading combination terms and articles on climate change and infectious diseases from 2009 to 2018.

The high-frequency terms were classified into four clusters for the first decade and five for the second decade using the biclustering analysis, as presented in Figure 3 and Figure 4. These two figures also show the mountain and matrix visualization of these terms. In the mountain visualization, each 3D peak labeled by the cluster number contains a cluster of terms, of which the location on the plane, volume, height, and color are used to portray information about a cluster. The distance between two peaks on the plane reflects the relative similarity of two clusters. There is positive correlation between the peak’s height and the cluster’s internal similarity. The volume of a peak is positively correlated with the number of terms classified into a cluster. In addition, the peak’s color represents the internal standard deviation of a cluster’s terms. Blue represents a high internal standard deviation of the objects inside, whereas red represents a low internal standard deviation. In the matrix visualization, the high-frequency terms are listed on the right side. The number before each term represents its serial number (See Tables S2 and S3). The top and left hierarchical trees display the relationships among the included articles and those among the high-frequency terms, by which the themes of all of the clusters have been able to identify and summarize, and insights into the representative articles of each cluster could be attained as well. The hotspots of climate change and infectious diseases revealed by the cluster analysis of high-frequency terms during the two periods are presented in Table 2.

### 3.3. Trends of Research Themes

A strategic diagram can generally represent the structure of a research field, in which all of the research hotspots are placed in the four quadrants of the coordinate graph, based on the values of the centrality and density, thus describing the research status and trend of each hotspot. The density is used to determine the closeness of the terms within each cluster of hotspots. It represents the self-sustainability of each cluster of hotspot, i.e., stability [41]. The centrality spot measure the closeness between the terms of each cluster and those in the other cluster, indicating the degree of mutual influence of one cluster of a hotspot and the other clusters. The greater the centrality of one cluster of hotspot, the more central it tends to be in the entire research field [42]. Therefore, the clusters in Quadrant 1 are the relative core and stable themes (strongly connected with other clusters and having intense internal relationships). The clusters located within Quadrant 2 represent peripheral but already well-developed themes. The clusters in Quadrant 3 are both peripheral and unstable. The clusters in Quadrant 4 are central but not stable, yet they are becoming mature or are vanishing to some extent [39]. Typically, analyses are the most interested in the new and exciting topics in Quadrant 4.

**Figure 5:**
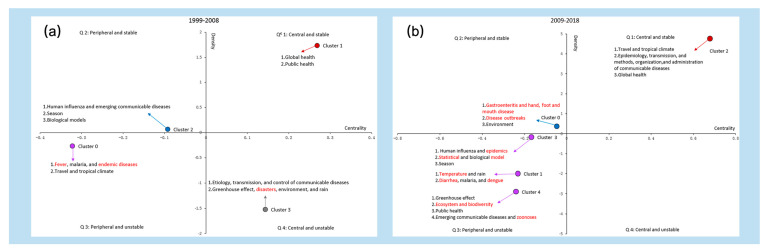
Strategic diagrams of hotspots in research output on climate change and infectious diseases from 1999 to 2008 and from 2009 to 2018. (**a**) Strategic diagrams of hotspot from 1999 to 2008; (**b**) strategic diagrams of hotspots from 2009 to 2018. ^€^ Q stands for quadrant. Clusters inside each strategic diagram refer to the clustering results shown in Table 2. The descriptions indicated by arrows are the hotspots of each cluster. The words in the red font represent the exclusive hotspot research topics in the first decade or the second decade.

Based on the results of the co-word biclustering analysis and the co-occurrence matrix of high-frequency terms, we calculated the centrality and density of each cluster and drew two strategic diagrams on research hotspots for the two decades. Then, we analyzed the basic framework, alteration, and trend of research hotspots on climate change and infectious diseases in the world (Figure 5).

Four clusters from 1999 to 2008 are scattered in the four quadrants, i.e., Cluster 1 in Quadrant 1, Cluster 2 in Quadrant 2, Cluster 0 in Quadrant 3, and Cluster 3 in Quadrant 4. Cluster 1, global and public health, is considered as the motor theme (of which centrality and density are both high). In the decade from 2009 to 2018, Cluster 2 lies in Quadrant 1, Cluster 0 in Quadrant 2, and the rest of three clusters (Clusters 1, 3, and 4) lie in Quadrant 3, while no cluster is in Quadrant 4. The contrast of the clusters and their positions in the strategic diagrams between the two 10-year periods can also be visualized in Figure 5, showing the trend and alteration of the hot research themes.

## 4. Discussion

In the context of global warming, academic communities have paid increasing attention to the direct and indirect effects of climate change on the appearance and spread of infectious diseases. The relationship between climate change and infectious diseases has become an important research field, which requires the systematic analysis of the knowledge structure and theme trends. In this study, the bibliometric analysis, co-word biclustering analysis, and strategic diagram on the scientific productions from the quantitative and content’s points of view were integrated to investigate the knowledge structure and evolution of this field in the past 20 years.

### 4.1. Principal Findings

To the authors’ knowledge, for the first time, an evaluation is reported on the research status and trend of climate change and infectious diseases around the world in the last two decades. The quantitative study clearly shows the exponentially rapid growth of the relevant publications, focusing on the topic of climate change and infectious diseases from 1999 to 2018. The articles published in the last decade (2009–2018) are nearly threefold of those in the previous decade (1999–2008).

According to the summarized distribution data of journals, the number of journals is almost twice as much in the last decade as in the previous decade, demonstrating that more and more journals began to publish articles regarding climate change and infectious diseases. It is also notable that comprehensive journals, for example, *Nature* and *PLoS One*, have shown interest in collecting papers in this field, confirming that the research on climate change-related infectious diseases had been supported by a wide range of subject categories. In the last two decades, however, the publication places of relevant journals were mainly developed countries or regions, such as The United States, England, Switzerland, and France.

As expected, this study provided some hints of the recent research hotspots in the field of climate change and infectious diseases. A co-word biclustering analysis was performed to reveal the research hotspots in the field, where there were four big clusters (nine subclass topics) and five big clusters (15) found in 1999 to 2008 and 2009 to 2018, respectively. Some topics, such as travel and tropic climate, global health, public health, environment, rain, biological model, and the greenhouse effect, have always been interesting for researchers. Human influenza, malaria, and emerging communicable diseases are the consistently concerned infectious diseases. From 1999 to 2008, hotspots like disasters, fever, and endemic diseases were not the same hotspots from 2009 to 2018. In the last ten years, researchers have begun to pay attention to some new hotspots, e.g., statistical model, diseases outbreaks, ecosystem, and biodiversity (Table 2 and Figure 5). Specifically, research on statistical models have become a hotspot, probably due to the development of data science, as well as new statistical methodology, such as deep learning, and their recent application in medicine [43]. Meanwhile, gastroenteritis; hand, foot, and mouth disease; diarrhea; dengue; and zoonoses are new foci among the research field of climate-sensitive communicable diseases. Hand, foot, and mouth disease, in particular, is a type of communicable disease emerging in recent decades. Although occurring much later in some countries like China, where it was originally seen in 1981, this disease is very prevalent, and often leads to outbreaks among children [44,45]. Therefore, researchers have begun to carry out related studies from multiple perspectives. In the aspect of its correlation with climate, a considerable amount of journal articles (32 articles) have been published within the last decade, according to the analysis of this study.

In parallel, the strategic diagram was employed to interpret the trends in themes during the two periods. Global health locates in Quadrant 1 all the time, indicating it is mature, but is indeed the eternal core theme of the whole body of literature. The topic on malaria is always in Quadrant 3, demonstrating that it is neither mature nor a core topic in the whole related field, and thus needs further investigation.

During the second decade (2009–2018), “Travel and tropical climate” within Quadrant 1, previously considered as the undeveloped and peripheral theme, progressed well and become the core of the relevant field. New hotspots of “gastroenteritis and hand, foot, and mouth disease” and “disease outbreaks” have become mature in the last ten years, although they are still on the edge of the whole field. Other new hotspots, like “Statistical model”, “Diarrhea”, “Dengue”, “Ecosystem and biodiversity”, and “Zoonoses”, are far from the research core, and do not connect closely with other subfield studies within the overall research network, and thus are neither mature nor the central topics in this field. They need more attention from researchers. However, research on some meteorological factors, such as “Greenhouse effect” and “Rain”, remain unstable or undeveloped, and have shifted from the central to the edge of the whole research field.

### 4.2. Limitations and Future Work

This present study has several potential limitations, which might encourage further research efforts. These include the research output of the target field being only represented by the publications in a single database. Bias could arise in terms of an underestimation or unbalanced estimation of each subfield. However, PubMed, as used in this study, is a world-renowned authoritative bibliographic database for biomedicine, from which the relevant literature could represent the research status on climate-related infectious diseases well, to certain degree. The second potential limitation is that as climate change and infectious diseases are closely related to national security and defense, due to their direct or indirect military applications, it is possible that part of the publications were not open to the public research community, thus they could not be included in the present bibliometric study for analysis. However, based on the open publications, the tendency and hotspots in the field can still be drawn. We plan to utilize diverse databases for further analyses in future studies.

## 5. Conclusions

While there is rising global attention to climate change, there is also increased research productivity in the field of climate change-related infectious diseases, which has been supported by a wide range of subject categories. At present, some significant public health challenges (global health, travel and tropical climate, etc.) are at the center of the whole target research network. “Statistical model”, “Diarrhea”, “Dengue”, “Ecosystem and biodiversity”, and “Zoonoses” were considered as emerging hotspots during the last ten years, but they still need more attention for further development. The present study provides the academic community and policymakers with baseline information in this field. Additionally, it provides a framework of the bibliometric analysis, co-word biclustering analysis, and strategic diagram on the scientific productions from a quantitative and contents points of view. The framework can assist the researchers to clarify the history, development, and trend in themes in the target field.

## Figures and Tables

**Table 1:** Most active journals of publications on climate change and infectious diseases in PubMed (1999–2008 and 2009–2018).

Period	Rank	Most Active Journals	Languages	Publication Places	Number of Publications	Percentage (%)
1999–2008	1	*Emerging Infectious Diseases*	English	United States	11	2.99
	2	*Journal of Travel Medicine*	English	England	9	2.45
	3	*Annals of the New York Academy of Sciences*	English	United States	6	1.63
	4	*International Journal of Health Geographics*	English	England	6	1.63
	5	*Medecine Tropicale: Revue du Corps de Sante Colonial*	French	France	6	1.63
	6	*Proceedings of the National Academy of Sciences of the United States of America*	English	United States	5	1.36
	7	*Epidemiology and Infection*	English	England	5	1.36
	8	*Revue Scientifique et Technique (International Office of Epizootics)*	English, French, Spanish	France	5	1.36
	9	*Nature*	English	England	5	1.36
	10	*New South Wales Public Health Bulletin*	English	Australia	5	1.36
	Total				63	17.12
2009–2018	1	*PLoS One*	English	United States	64	5.93
	2	*International Journal of Environmental Research and Public Health*	English	Switzerland	31	2.87
	3	*Epidemiology and Infection*	English	England	25	2.32
	4	*BMC Infectious Diseases*	English	England	23	2.13
	5	*PLoS Neglected Tropical Diseases*	English	United States	23	2.13
	6	*Przeglad Epidemiologiczny*	English, Polish	Poland	18	1.67
	7	*Proceedings of the National Academy of Sciences of the United States of America*	English	United States	18	1.67
	8	*Communicable Diseases Intelligence Quarterly Report*	English	United States	17	1.58
	9	*Emerging Infectious Diseases*	English	United States	17	1.58
	10	*Global Health Action*	English	United States	16	1.48
	Total				252	23.35

**Table 2:** Hotspots of climate change and infectious diseases explored by biclustering analysis of high-frequency major MeSH/subheading combination terms in 1999–2008 and 2009–2018.

Period	Cluster	Term * No.	Cluster Analysis
1999–2008	0	22, 25, 5, 9, 26	1. Fever, malaria, and endemic diseases; 2. Travel and tropical climate
	1	17, 19, 2, 10, 12	1. Global health 2. Public health
	2	16, 13, 6, 11, 4, 15	1. Human influenza and emerging communicable diseases 2. Season 3. Biological models
	3	21, 20, 18, 8, 3, 14, 1, 23, 24, 7	1. Etiology, transmission, and control of communicable diseases 2. Greenhouse effect, disasters, environment, and rain
2009–2018	0	36, 4, 10, 15, 19, 35	1. Gastroenteritis and hand, foot, and mouth disease 2. Disease outbreaks 3. Environment
	1	18, 32, 39, 30, 22, 7, 11	1. Temperature and rain 2. Diarrhea, malaria, and dengue
	2	24, 12, 21, 31, 20, 5, 2, 1, 28	1. Travel and tropical climate 2. Epidemiology, transmission, and methods, organization, and administration of communicable diseases 3. Global health
	3	34, 23, 17, 8, 3, 9, 27, 29	1.Human influenza and epidemics 2. Statistical and biological model 3. Season
	4	26, 25, 13, 37, 38, 6, 16, 14, 33	1. Greenhouse effect 2. Ecosystem and biodiversity 3. Public health 4. Emerging communicable diseases and zoonoses

* Term refers to the high-frequency major MeSH/subheading combination term.

## Supplementary Materials

The following are available online at https://www.mdpi.com/1660-4601/17/14/5228/s1: Table S1: Search strategy for retrieval of literature on climate change and infectious diseases from PubMed; Table S2: High-frequency major MeSH/Subheading combination terms from the included articles on climate and infectious diseases in 1999–2008; Table S3: High-frequency major MeSH/Subheading combination terms from the included articles on climate and infectious diseases in 2009–2018.
