# Supplementary material for: Global Research Output and Theme Trends on Climate Change and Infectious Diseases: A Restrospective Bibliometric and Co-Word Biclustering Investigation of Papers Indexed in PubMed (1999–2018)
Source: Int J Environ Res Public Health. 2020 Jul 20;17(14):5228. doi: 10.3390/ijerph17145228 (PMC7400491; doi:10.3390/ijerph17145228)
Supplement: Supplementary file 1 [file ijerph-17-05228-s001.zip › Supplementary files/Table S2.docx]

**Table S2.** High-frequency major MeSH/Subheading combination terms from the included articles on climate and infectious diseases in 1999-2008

| **No.** | **Major MeSH/Subheading combination terms** | **Frequency** | **Proportion of frequency (%)** | **Cumulative percentage (%)** |
| --- | --- | --- | --- | --- |
| 1 | Communicable Diseases / epidemiology | 66 | 4.80 | 4.80 |
| 2 | Climate | 51 | 3.71 | 8.51 |
| 3 | Greenhouse Effect | 40 | 2.91 | 11.42 |
| 4 | Seasons | 34 | 2.47 | 13.89 |
| 5 | Travel | 28 | 2.04 | 15.93 |
| 6 | Communicable Diseases, Emerging / epidemiology | 26 | 1.89 | 17.82 |
| 7 | Disease Outbreaks | 25 | 1.82 | 19.64 |
| 8 | Communicable Diseases / transmission | 18 | 1.31 | 20.95 |
| 9 | Tropical Climate | 16 | 1.16 | 22.11 |
| 10 | Global Health | 16 | 1.16 | 23.27 |
| 11 | Disease Outbreaks / statistics & numerical data | 14 | 1.02 | 24.29 |
| 12 | Weather | 13 | 0.95 | 25.24 |
| 13 | Population Surveillance | 12 | 0.87 | 26.11 |
| 14 | Disease Vectors | 11 | 0.80 | 26.91 |
| 15 | Models, Biological | 10 | 0.73 | 27.64 |
| 16 | Influenza, Human / epidemiology | 10 | 0.73 | 28.36 |
| 17 | Health Status | 7 | 0.51 | 28.87 |
| 18 | Communicable Disease Control | 7 | 0.51 | 29.38 |
| 19 | Public Health | 7 | 0.51 | 29.89 |
| 20 | Communicable Diseases / etiology | 6 | 0.44 | 30.33 |
| 21 | Disasters | 6 | 0.44 | 30.76 |
| 22 | Endemic Diseases | 6 | 0.44 | 31.20 |
| 23 | Environment | 6 | 0.44 | 31.64 |
| 24 | Rain | 6 | 0.44 | 32.07 |
| 25 | Fever / etiology | 6 | 0.44 | 32.51 |
| 26 | Malaria / epidemiology | 6 | 0.44 | 32.95 |
