# Supplementary material for: Global Research Output and Theme Trends on Climate Change and Infectious Diseases: A Restrospective Bibliometric and Co-Word Biclustering Investigation of Papers Indexed in PubMed (1999–2018)
Source: Int J Environ Res Public Health. 2020 Jul 20;17(14):5228. doi: 10.3390/ijerph17145228 (PMC7400491; doi:10.3390/ijerph17145228)
Supplement: Supplementary file 1 [file ijerph-17-05228-s001.zip › Supplementary files/Table S3.docx]

**Table S3.** High-frequency Major MeSH/Subheading combination terms from the included articles on climate and infectious diseases in 2009-2018

| **No.** | **Major MeSH/Subheading combination terms** | **Frequency** | **Proportion of frequency (%)** | **Cumulative percentage (%)** |
| --- | --- | --- | --- | --- |
| 1 | Climate Change | 192 | 4.96 | 4.96 |
| 2 | Communicable Diseases / epidemiology | 180 | 4.65 | 9.61 |
| 3 | Seasons | 97 | 2.51 | 12.11 |
| 4 | Disease Outbreaks | 64 | 1.65 | 13.77 |
| 5 | Communicable Diseases / transmission | 58 | 1.50 | 15.26 |
| 6 | Communicable Diseases, Emerging / epidemiology | 56 | 1.45 | 16.71 |
| 7 | Climate | 54 | 1.39 | 18.10 |
| 8 | Models, Biological | 50 | 1.29 | 19.40 |
| 9 | Influenza, Human / epidemiology | 41 | 1.06 | 20.45 |
| 10 | Weather | 38 | 0.98 | 21.44 |
| 11 | Dengue / epidemiology | 34 | 0.88 | 22.31 |
| 12 | Tropical Climate | 34 | 0.88 | 23.19 |
| 13 | Public Health | 33 | 0.85 | 24.04 |
| 14 | Disease Vectors | 33 | 0.85 | 24.90 |
| 15 | Hand, Foot and Mouth Disease / epidemiology | 32 | 0.83 | 25.72 |
| 16 | Zoonoses / epidemiology | 29 | 0.75 | 26.47 |
| 17 | Disease Outbreaks / statistics & numerical data | 27 | 0.70 | 27.17 |
| 18 | Temperature | 27 | 0.70 | 27.87 |
| 19 | Models, Theoretical | 26 | 0.67 | 28.54 |
| 20 | Global Health | 24 | 0.62 | 29.16 |
| 21 | Communicable Disease Control / methods | 23 | 0.59 | 29.75 |
| 22 | Malaria / epidemiology | 22 | 0.57 | 30.32 |
| 23 | Models, Statistical | 22 | 0.57 | 30.89 |
| 24 | Travel | 21 | 0.54 | 31.43 |
| 25 | Ecosystem | 21 | 0.54 | 31.97 |
| 26 | Greenhouse Effect | 21 | 0.54 | 32.52 |
| 27 | Influenza, Human / prevention & control | 19 | 0.49 | 33.01 |
| 28 | Health Status | 18 | 0.46 | 33.47 |
| 29 | Population Surveillance | 17 | 0.44 | 33.91 |
| 30 | Diarrhea / epidemiology | 16 | 0.41 | 34.32 |
| 31 | Communicable Disease Control / organization & administration | 15 | 0.39 | 34.71 |
| 32 | Rain | 15 | 0.39 | 35.10 |
| 33 | Communicable Diseases, Emerging / transmission | 14 | 0.36 | 35.46 |
| 34 | Epidemics | 13 | 0.34 | 35.80 |
| 35 | Environment | 13 | 0.34 | 36.13 |
| 36 | Gastroenteritis / epidemiology | 12 | 0.31 | 36.44 |
| 37 | Biodiversity | 12 | 0.31 | 36.75 |
| 38 | Communicable Diseases, Emerging / prevention & control | 12 | 0.31 | 37.06 |
| 39 | Water Microbiology | 12 | 0.31 | 37.37 |
