# Supplementary material for: Global Research Output and Theme Trends on Climate Change and Infectious Diseases: A Restrospective Bibliometric and Co-Word Biclustering Investigation of Papers Indexed in PubMed (1999–2018)
Source: Int J Environ Res Public Health. 2020 Jul 20;17(14):5228. doi: 10.3390/ijerph17145228 (PMC7400491; doi:10.3390/ijerph17145228)
Supplement: Supplementary file 1 [file ijerph-17-05228-s001.zip › Supplementary files/Table S1.doc]

**Table S1.** Search strategy for retrieval of literature on climate change and infectious diseases from PubMed

| **Search** | **Query** |
| --- | --- |
| #1 | "Meteorological Concepts"[Mesh] |
| #2 | "Climate"[Mesh] |
| #3 | "Climate Change"[Mesh] |
| #4 | "Weather"[Mesh] |
| #5 | meteorological[tiab] OR meteorologica[tiab] OR meteorologic[tiab] OR meteorologically[tiab] |
| #6 | weather[tiab] |
| #7 | climate[tiab] |
| #8 | "Communicable Diseases"[Mesh] |
| #9 | infectious diseases[tiab] OR infectious disease[tiab] OR communicable diseases[tiab] OR communicable disease[tiab] |
| #10 | #1 OR #2 OR #3 OR #4 OR #5 OR #6 OR #7 |
| #11 | #8 OR #9 |
| #12 | #10 AND #11 |
